# Editorial: Reducing adverse effects of cancer immunotherapy

**DOI:** 10.3389/fimmu.2024.1368496

**Published:** 2024-02-01

**Authors:** Daniele Maria-Ferreira, Elizabeth Soares Fernandes, Cleber Machado-Souza, Carolina Silva Schiebel, Andressa Caroline Dos Santos Maia, Leonardo Vinícius Barbosa

**Affiliations:** ^1^ Instituto de Pesquisa Pelé Pequeno Príncipe, Faculdades Pequeno Príncipe, Curitiba, Brazil; ^2^ Programa de Pós-graduação em Biotecnologia Aplicada à Saúde da Criança e do Adolescente, Faculdades Pequeno Príncipe, Curitiba, Brazil

**Keywords:** immunotherapy side effects, immunotherapy toxicity, cancer treatment toxicity, immunotherapy tolerability, cancer treatment

Chemotherapy and radiotherapy are associated with various adverse effects. Appropriate management ensures compliance with treatment and, above all, the patient’s well-being. Immunotherapy has emerged as a new and promising option for cancer treatment. However, immunotherapy is also associated with immune-related adverse events. Thirteen articles have been published on this Research Topic.

Kovalenko et al. conducted a systematic review and meta-analysis of clinical trials investigating the toxicity profile of combined ICI therapy versus ICI + VEGFi. ICIs inhibit the excessive activation of immune checkpoint signaling pathways, while VEGFi acts by interfering with vascular endothelial growth factor or by blocking the VEGF receptor. The authors showed that TRAEs occurred more frequently in the ICT + VEGFi group and treatment discontinuations were attributed to these adverse events. The largest increase in TRAE effect size was seen for rash, hypertension, hypothyroidism and diarrhea, but other TRAEs such as nausea, anorexia and anemia were also common. The results suggest that combination therapy is directly associated with a higher risk of some TRAEs compared to monotherapy.

Qiu et al. evaluated the efficacy and clinical safety of trilaciclib, a CDK4/6 inhibitor that protects blood cell lines from chemotherapy, for the prevention of chemotherapy-induced myelosuppression through a meta-analysis. Only 4 randomized controlled trials of patients with small cell lung cancer or breast cancer were evaluated. The use of trilaciclib reduced the incidence and duration of severe neutropenia, the incidence of febrile neutropenia and anemia. The therapeutic use of erythropoiesis-stimulating agents, granulocyte colony-stimulating factors and erythrocyte transfusions was also reduced in patients treated with trilaciclib. Overall survival and progression-free survival were identical in the control group and the trilaciclib group. In summary, trilaciclib has an acceptable safety profile, does not interfere with chemotherapy, and effectively reduces the incidence of myelosuppression.

Three case reports and a systematic analysis of case reports were published. Wu et al. described a case of cardiotoxicity due to the use of Tislelizumab, a PD-1 inhibitor. The case involved a 59-year-old male patient with a history of non-small cell squamous cell carcinoma of the lung and coronary artery disease. After cardiotoxicity occurred, tislelizumab was discontinued and replaced with sugemalimab, a PD-L1 inhibitor, after which no further cardiac changes were observed. The authors assume that these results could contribute to the optimization of cancer immunotherapy.

Ohmura et al. reported a case of RS3PE after the use of nivolumab, a monoclonal antibody that targets the anti-PD1 receptor, for the treatment of gastric cancer. Despite the decrease in tumor markers and metastatic lymph node lesions, the patient showed symptoms of RS3PE syndrome, such as pain, edema, lymphocytic and macrophage infiltration in skin, and CD4+ or CD8+ T-cell infiltration in the perivascular area. Therapy with prednisolone, which inhibits inflammatory cells and suppresses the expression of inflammatory mediators by binding to glucocorticoid receptors, was initiated, and the patient was referred to supportive care. The authors suggest that the results will be useful to elucidate immune-related adverse events triggered by anti-PD-1 drugs.

Zhou et al. describes a case of tislelizumab-induced uretitis/cystitis in a male patient with thymic carcinoma. The patient was treated with tislelizumab, a humanized IgG4 monoclonal antibody with a strong affinity for PD-1 binding; paclitaxel-albumin, an antimicrotubule combined with albumin; and carboplatin, a second-generation platinum (II) complex that acts as an atypical alkylator, binds and cross-links the DNA, leading to breakage of the DNA strand during replication. The patient was hospitalized after suffering from severe abdominal and back pain that was not relieved by antibiotics and antispasmodics. The patient had dilation of the urinary tract and cystitis, which resolved after discontinuation of tislelizumab. The authors compare their case report with other cases of cystitis associated with immunotherapy and re-emphasize that the data presented may contribute to the diagnosis of unique immune-related adverse events.

Finally, Wang et al. performed a systematic analysis of case reports to evaluate evidence of ICI-associated myocarditis. A total of 113 publications from 106 patients were analyzed, with myocarditis occurring in 53.8% of cases and more than half of the cases being fatal or severe. The authors concluded that treatment of high-grade myocarditis associated with ICI use should be managed with strategies that include, for example, discontinuation of ICIs in conjunction with high-dose glucocorticoids. The information provided by the authors may assist in medical decision making.

Yang et al. investigated the possible association between aspirin, a non-steroid anti-inflammatory drug, use and irAEs in patients receiving immunotherapy. Information from the FAERS was used for this purpose. An association between aspirin use and an increased risk of irAEs was found in patients with lung cancer, mesothelioma, and pancreatic cancer, while there was a lower risk in patients with lymphoma. Major irAEs included anemia, myositis, colitis and others. Aspirin use was associated with a lower risk of skin rash, thyroiditis, and Stevens-Johnson syndrome. This information may be useful for future studies on individualized treatment plans.

In a retrospective study, Wang et al. investigated the effect and safety of prednisone, a steroidal anti-inflammatory drug, on persistent hematologic toxicity after CAR-T cell therapy, that is suggested to induce a T-cell response against antigen-expressing cells, in 17 patients with acute B-cell lymphoblastic leukemia. Administration of prednisone at an initial dose of 0.5 mg/kg/day resulted in 100% recovery of blood counts and a complete recovery of 60 to 66.67%. Hematologic toxicities recurred in 6 patients after discontinuation of treatment. The median follow-up time was approximately 14 months, progression-free survival was 58.8% and overall survival was 64.7%. The authors suggest that treatment with prednisone could be an interesting option for hematologic toxicity due to treatment with CAR-T cells.

Li et al. retrospectively analyzed 12 medical records of patients with interstitial lung disease that occurred after taking antineoplastic drugs. The authors showed that DILD was triggered by different classes of drugs, with the use of ICIs accounting for approximately 66% of cases. The authors pointed out that DILD occurs mainly in male, elderly patients with lung cancer and that some specific measures and special care are needed to improve the prognosis of DILD.

Gutierrez et al. retrospectively investigated the effects of anti-CD20 maintenance on both responses to the SARS-CoV-2 vaccine and the incidence/severity of COVID-19. The monoclonal anti-CD20 antibodies can act via several mechanisms. A significantly increased risk of severe COVID-19 within the first 24 months after the last administration of anti-CD20 was observed. Neither vaccine response nor hypogammaglobulinemia had a significant impact on overall survival. The results suggest that anti-CD20 therapy impairs the serologic response to SARS-CoV-2 vaccines. However, certain measures, such as monitoring the intake of immunoglobulins or ensuring adequate immunization, may help to mitigate this effect.

In contrast to other studies, Gong et al. used a sophisticated machine learning approach to develop a model to predict individual risk of ICI-induced IRP. Retrospective data from 48 patients with IRP and 142 without IRP who were treated with ICIs were included. Eleven predictors were used, including history of lung disease and cancer stage. The model validation showed good discrimination and acceptable calibration ability, with AUC values of 0.81, an average precision of 0.76, a scaled Brier score of 0.31, and a Spiegelhalter z of −0.29. An online risk calculator was developed, and the authors concluded that the prediction model is accurate and can be used in clinical practice.

Huang et al. also used a machine learning approach, but to identify risk factors that contribute to the development of CIM in patients with gastrointestinal cancer. Frequency and severity of CIM were analyzed in 328 patients. The authors showed significant correlations between the incidence of mucositis and gender, the number of chemotherapy cycles and the administration of platinum-based drugs, that cross-link DNA strands and inhibit DNA synthesis and function, and irinotecan, that inhibits the action of topoisomerase I by interfering with the moving replication fork and causing replication arrest and lethal double-strand breaks in DNA. A positive correlation between the occurrence of diarrhea and surgical history, treatment with irinotecan and the use of probiotics (p = 0.037, 0.021 and 0.035, respectively) and a negative correlation with platinum-based treatment (p = 0.026) were found. Thus, the authors have successfully completed the development and implementation of the prediction model.

Park et al. used HUVECs cells and male C57BL/6 mice to test the ability of CU06-1004, a blocker of endothelial dysfunction, to inhibit endothelial permeability induced by HDIL-2, a recombinant form of human IL-2 that binds to the IL-2 receptor and activates various signaling pathways. Treatment with CU06-1004 promoted the maintenance of cellular stability and prevented HDIL-2-induced vascular leakage *in vitro* and *in vivo*, respectively. Co-administration of HDIL-2 and CU06-1004 effectively reduced tumor growth in the B16F10 mouse model. In conclusion, the authors emphasize that CU06-1004 prevents vascular leakage syndrome and has anti-cancer potential.

The treatment of cancer is still accompanied by various toxic off-target effects. Further research is essential to improve understanding and ensure optimal treatment with minimal discomfort for patients. This Research Topic has provided new and pertinent information that will make a valuable contribution to the advancement of knowledge in this field. We would like to thank all the authors, reviewers and editors who have contributed to this Research Topic.

## Author contributions

DM-F: Conceptualization, Writing – original draft, Writing – review & editing. EF: Conceptualization, Writing – original draft, Writing – review & editing. CMS: Conceptualization, Writing – original draft, Writing – review & editing. CSS: Writing – original draft. AM: Writing – original draft. LB: Writing – original draft.

